# Epidemiological Society

**Published:** 1897-03-06

**Authors:** 


### EPIDEMIOLOGICAL SOCIETY.

At the last meeting held on February 19th a paper was read by Dr. John C. McVail, the subject being "The Report of the Royal Commission on Vaccination: A Review of the Dissentients' Statement." We cannot pretend to publish even an abstract of so ponderous a paper. It may be possible, however, to give an outline of the criticism of the author upon the final recommendations given by the dissentients. Instead, he says, of vaccination being abolished it is to remain optional. All its machinery is to be left intact, and efforts to secure it are not in the least to be prohibited, but are only to be limited to "moral influence." National funds are to continue to be expended in giving parents the opportunity of obtaining vaccination, or, in the view of the dissentients, of exposing their children to risks (which in the houses of the poor are declared to be unavoidable) of vaccinal disease and vaccinal death, at the same time that the need for vaccination as a protection shall have been removed by means of compulsory isolation! The whole attitude is extraordinary, and an explanation may well be sought for. It seems to me, he says, that no explanation sufficient to meet the case can be found excepting this, that the dissentients have more faith in vaccination and less faith in isolation than they are willing to admit even to themselves. They see how in unvaccinated Gloucester small-pox broke out in a manner which foreshadows what may be looked for all over England if vaccination falls into abeyance. They want to be prepared for such an emergency, and, in addition to sanitation and hospitals, this preparation is to consist in the maintenance of all the machinery for vaccination. Government is to be able to supply calf lymph without stint. Officers are to be ready for carrying out vaccination and revaccination. Funds raised by assessment are to be legally available to meet all expenditure. In short, instead of vaccination systematically carried out, there is to be panic vaccination—vaccination in the immediate presence of small-pox, and under circumstances in which the rigid and scrupulous care which the Commission recommends will run risk of being neglected.

It is a strange and interesting thing that, while the Royal Commission, after proving up to the hilt the entire efficacy of vaccination, failed to recommend its compulsory employment, the dissentients, after doing their best throughout, both during the inquiry and in their statement, to discredit vaccination, should end by advising its retention as an official and recognised means of prophylaxis, supported at the public expense.

